# SirT3 activates AMPK-related mitochondrial biogenesis and ameliorates sepsis-induced myocardial injury

**DOI:** 10.18632/aging.103644

**Published:** 2020-07-28

**Authors:** Ting Xin, Chengzhi Lu

**Affiliations:** 1 Department of Cardiology, Tianjin First Central Hospital, Tianjing 300192, P.R. China

**Keywords:** SirT3, AMPK, septic cardiomyopathy, mitochondrial biogenesis

## Abstract

Sirtuin-3 (SirT3) and AMPK stimulate mitochondrial biogenesis, which increases mitochondrial turnover and cardiomyocyte regeneration. We studied the effects of SirT3, AMPK, and mitochondrial biogenesis on sepsis-induced myocardial injury. Our data showed that after treating cardiomyocytes with lipopolysaccharide, SirT3 and AMPK levels decreased, and this was followed by mitochondrial dysfunction and cardiomyocyte death. Overexpression of SirT3 activated the AMPK pathway and improved mitochondrial biogenesis, which is required to sustain mitochondrial redox balance, maintain mitochondrial respiration, and suppress mitochondrial apoptosis. Inhibition of mitochondrial biogenesis abolished SirT3/AMPK-induced cardioprotection by causing mitochondrial damage. These findings indicate that SirT3 reduces sepsis-induced myocardial injury by activating AMPK-related mitochondrial biogenesis.

## INTRODUCTION

Acute cardiac injury caused by septic shock is a common cardiovascular complication in critically ill patients [1]. In septic shock, the myocardium is injured due to a variety of causes, including severe infection, anoxia, ischemia, trauma, and surgery, resulting in high morbidity and mortality [2]. Unfortunately, no effective treatment exists for septic cardiomyopathy. Thus, studying the molecular pathogenesis of acute myocardial injury may provide an effective target for the early diagnosis and treatment of septic cardiomyopathy.

Recently, studies of sepsis-induced myocardial injury have found that excessive reactive oxygen species (ROS) production caused by mitochondrial damage results in increased oxidative stress, leading to cell death and tissue damage [3]. Studies have also found that early treatment with ROS scavengers is an effective therapy for septic cardiomyopathy [4, 5]. At the molecular level, ROS-induced cardiomyocyte oxidative stress promotes protein oxidation and lipid peroxidation [6], which reduce cardiomyocyte contractility and thus decrease heart pump function, leading to low perfusion of distant organs or tissues [7]. More severely, oxidative stress triggers cardiomyocyte death through apoptosis, necroptosis, and necrosis [8]. Mitochondria are the main source of ROS and are vulnerable to oxidative stress [9]. Therefore, protection of mitochondria is vital to suppress oxidative stress.

Mitochondrial biogenesis is the process of mitochondrial degradation and regeneration [10]. It is regulated by several nuclear genes that control mitochondrial DNA synthesis and protein expression, such as peroxisome proliferator-activated receptorγcoactivator-1α (PGC1α) [11]. PGC1α, a member of the transcriptional coactivator family PGC-1, primarily exists in high-energy-demand tissues and organs, including the heart, controlling both mitochondrial biogenesis and energy metabolism [12, 13]. PGC-1α expression is activated by a variety of stresses such as oxidative stress, exercise training, myocardial ischemia-reperfusion injury, myocardial infarction, and cardiac fibrosis [14, 15]. Elevated PGC-1α expression is correlated with increased mitochondrial biogenesis, which sustains mitochondrial quality and quantity [16]. Conversely, knockout of PGC-1α decreases cardiac function, possibly due to mitochondrial turnover arrest and cardiomyocyte mitochondrial death [17]. At the molecular level, PGC-1α transcription and activity are controlled by the AMPK pathway [18]. The protective function of AMPK on diabetic cardiomyopathy, cardiac ischemia-reperfusion injury, cardiac remodeling, and inflammation-related myocardial damage has been widely studied [19, 20]. Considering the beneficial effects exerted by AMPK and PGC-1α on mitochondrial homeostasis and cardioprotection, determining their effect on sepsis-related myocardial injury and mitochondrial damage is important.

Sirtuin-3 (SirT3) is a nuclear NAD^+^-dependent histone deacetylase that regulates mitochondrial oxidative stress and bioenergetics [21]. SirT3 increases the expression of genes related to mitochondrial DNA repair through deacetylation, including *NEIL1*, *NEIL2*, *OGG1*, *MUTYH*, *APE1*, and *LIG3* [22]. SirT3 also inhibits mitochondria-mediated apoptosis in cardiomyocyte ischemia-reperfusion injury, and this effect has been associated with activation of the AMPK pathway [23]. Anti-senescence [24], anti-fibrosis [25], and anti-inflammation [26] effects of SirT3 have also been demonstrated. Increased SirT3 has been shown to prevent apoptosis [23], oxidative stress [27], mitochondrial fission [28], unfolded protein response [29], and metabolic reprogramming [30] in many cardiovascular disorders. However, there is a lack of evidence demonstrating the effect of SirT3 on septic cardiomyopathy. The aim of our study is to determine whether SirT3 protects cardiomyocytes against septic shock by sustaining mitochondrial biogenesis via the AMPK pathway.

## RESULTS

### SirT3 and AMPK are downregulated in LPS-induced septic cardiomyopathy

**Figure 1:**
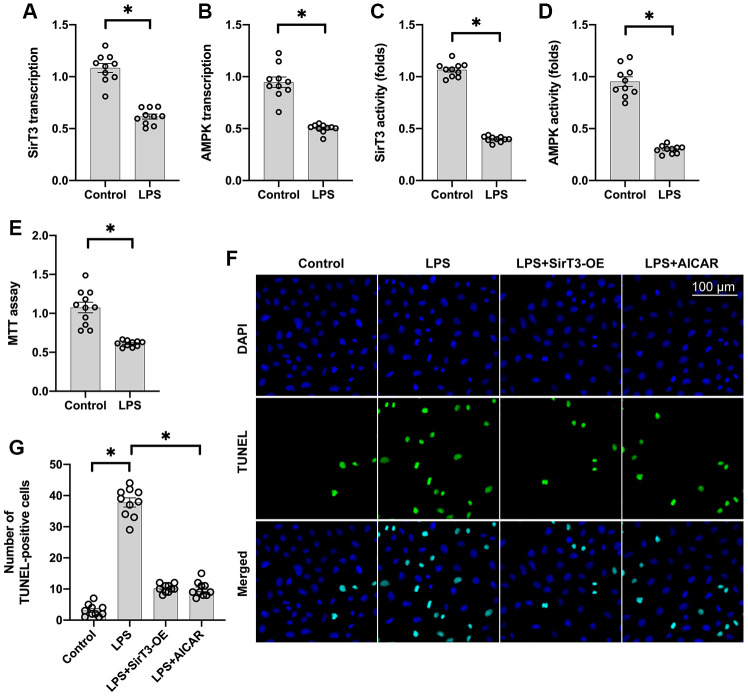
**SirT3 and AMPK are downregulated in response to LPS-induced septic cardiomyopathy.** (**A**, **B**) RNA was isolated, and the transcription of SirT3 and AMPK was remeasured. (**C**, **D**) ELISA was used to analyze the activities of SirT3 and AMPK in response to LPS treatment. (**E**) MTT assay was used to detect cardiomyocyte viability under LPS treatment. (**F**, **G**). TUNEL staining for apoptotic cardiomyocytes. Lentivirus-loaded SirT3 (SirT3-OE) and AMPK agonist (AICAR) were incubated with cardiomyocytes in the presence of LPS. Then, the number of TUNEL-positive cardiomyocytes was determined. *P < 0.05.

Lipopolysaccharide (LPS) was used to establish the septic cardiomyocyte model in vitro. Then, RNA was isolated to analyze the alterations of SirT3 and AMPK in cardiomyocytes. As shown in Figure 1A, 1B, compared to the control group, SirT3 was significantly downregulated. A decrease in AMPK transcription was also seen. These data indicate that LPS suppresses SirT3 and AMPK transcription. In addition, ELISA assay demonstrated that SirT3 and AMPK activity was significantly downregulated in cardiomyocytes after exposure to LPS (Figure 1C, 1D), suggesting that LPS may inhibit SirT3 and AMPK protein expression. Cardiomyocyte viability was also reduced in response to LPS treatment (Figure 1E). To determine whether SirT3 and AMPK inactivation contributes to LPS-mediated cardiomyocyte death, lentivirus-loaded SirT3 (SirT3-OE) and AMPK agonist (AICAR) were incubated with cardiomyocytes in the presence of LPS. Then, cardiomyocyte viability was measured using the TUNEL assay. As shown in Figure 1F, 1G, approximately 3% of cardiomyocytes were TUNEL positive under normal condition, whereas this rate increased to approximately 41% after exposure to LPS. Treatment with either Sirt3-OE or AICAR drastically suppressed the rate of TUNEL-positive cardiomyocytes. Together, our results indicate that SirT3 and AMPK are inhibited by LPS, leading to cardiomyocyte damage.

### Overexpression of SirT3 or activation of AMPK reduces LPS-induced cardiomyocyte dysfunction

**Figure 2:**
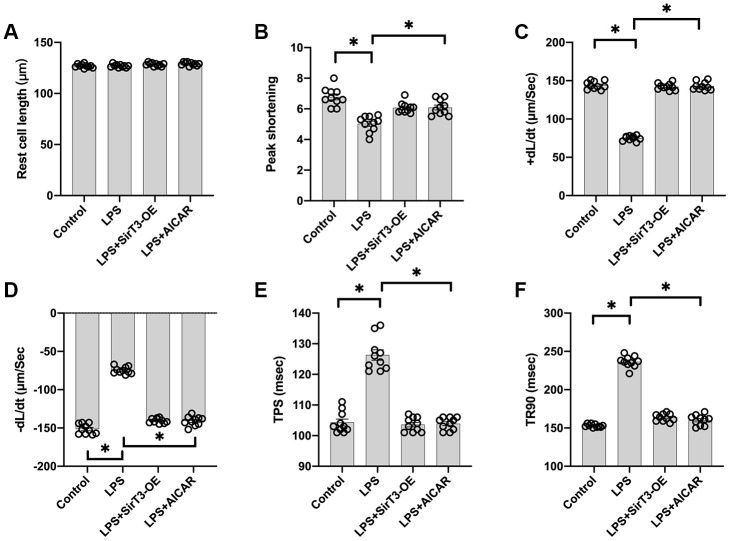
**Overexpression of SirT3 or activation of AMPK attenuates LPS-mediated cardiomyocyte dysfunction.** (**A**–**F**) Cardiomyocyte contractility in response to LPS treatment. Lentivirus-loaded SirT3 (SirT3-OE) and AMPK agonist (AICAR) were incubated with cardiomyocyte in the presence of LPS. +dL/dt is the maximal velocity of shortening. −dL/dt is the maximal velocity of relengthening. TPS, time to peak shortening; TR90, time to 90% relengthening. *P < 0.05.

Cardiomyocyte contractile properties determine cardiac function [31]. Previous studies have reported that septic cardiomyopathy is characterized by depressed myocardial ejection function, which results in lower organ blood perfusion [32, 33]. In our study, a single cardiomyocyte was isolated, and cardiomyocyte mechanical features were observed. As shown in Figure 2A–2F, the resting cell length of the cardiomyocyte was similar in the presence or absence of LPS. In addition, SirT3-OE or AICAR treatment did not affect resting cell length. However, LPS treatment significantly reduced peak shortening in the cardiomyocyte, and this effect was improved by the addition of SirT3-OE or AICAR (Figure 2A–2F). Similarly, the cardiomyocyte maximal velocity of shortening (+dL/dt) was also suppressed by LPS, whereas SirT3-OE or AICAR treatment restored the +dL/dt (Figure 2A–2F). In addition to the maximal velocity of shortening, the maximal velocity of relengthening (−dL/dt), which is used to evaluate cardiomyocyte relaxation, was also compromised by LPS treatment and reversed to near-normal levels with Sirt3-OE or AICAR treatment (Figure 2A–2F). To quantify cardiomyocyte contractile and diastolic function, time to peak shortening (TPS) and time to 90% relengthening (TR90) were measured [34], as previously described. As shown in Figure 2A–2F, compared to the control group, both TPS and TR90 increased after LPS treatment, suggesting impaired cardiomyocyte contractile and diastolic capacities. However, SirT3-OE or AICAR treatment improved TPS and TR90 in cardiomyocytes that had been treated with LPS (Figure 2A–2F). Our results indicate that SirT3 and AMPK are important in sustaining cardiomyocyte mechanical properties in the presence of LPS.

### SirT3 and AMPK sustain mitochondrial function

**Figure 3:**
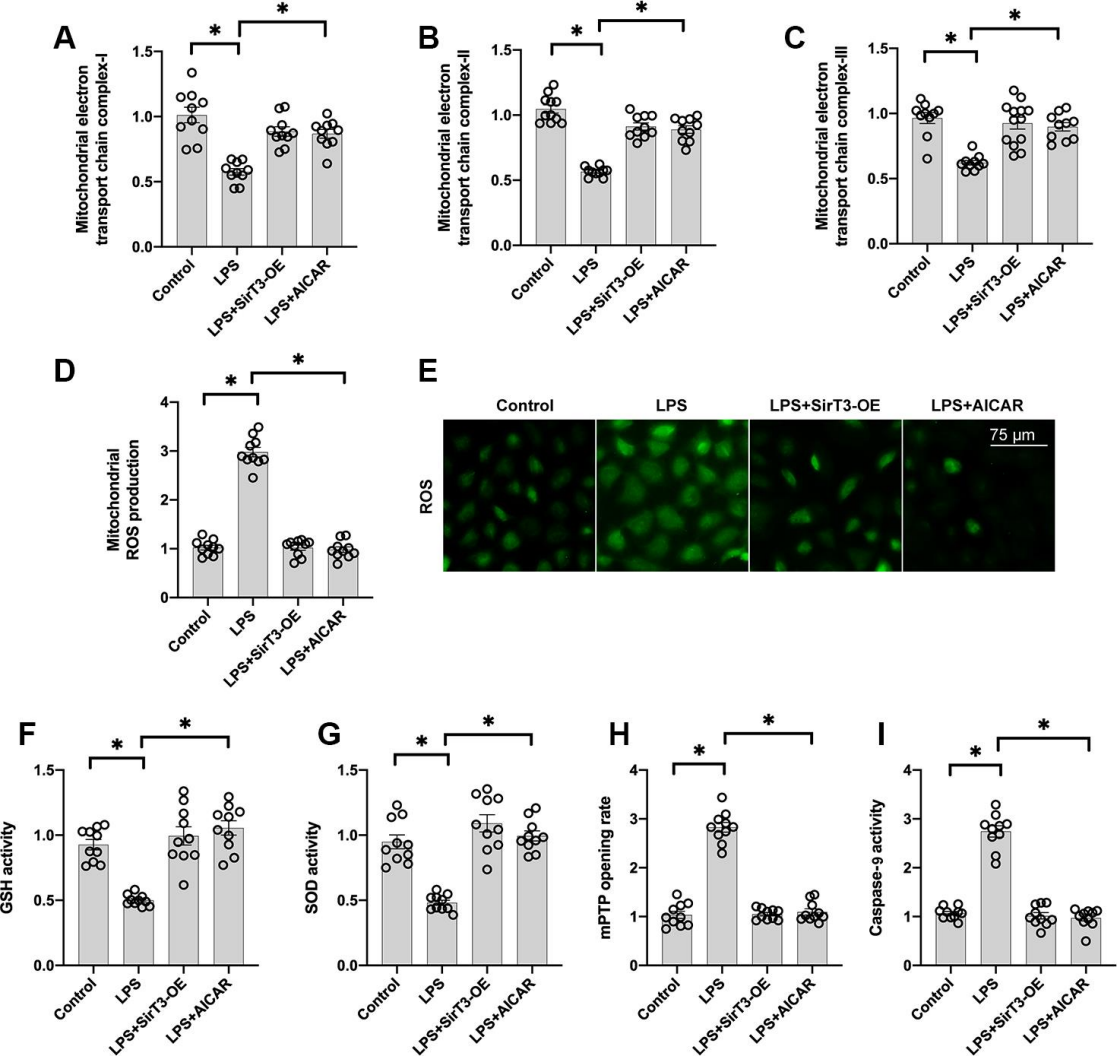
**SirT3 and AMPK sustain mitochondrial function.** (**A**–**C**) ELISA assay was used to analyze the activity of mitochondrial electron transport chain complexes in the presence of LPS. Lentivirus-loaded SirT3 (SirT3-OE) and AMPK agonist (AICAR) were incubated with cardiomyocytes before LPS treatment. (**D**–**E**) Immunofluorescence for mitochondrial ROS production. (**F**, **G**) GSH and SOD activity was measured using ELISA. (**H**) mPTP opening rate was measured in response to LPS treatment, SirT3-OE transfection, and AICAR supplementation. (**I**) ELISA was used to detect the activity of caspase-9. *P < 0.05.

At the subcellular level, SirT3 is primarily localized in mitochondria and sustains mitochondrial bioenergetics [35]. In addition, mitochondria-mediated ATP production is vital for cardiomyocyte contraction and relaxation [36]. Thus, we wanted to determine whether mitochondrial function is sustained by SirT3 and AMPK in LPS-treated cardiomyocytes. First, mitochondrial metabolism was determine by analyzing mitochondrial respiration. Compared to the control group, the activity of mitochondrial electron transport chain complexes was significantly downregulated by LPS treatment (Figure 3A–3C); this effect was reversed by SirT3-OE or AICAR treatment. Subsequently, due to impaired mitochondrial respiration, mitochondrial ROS production was significantly elevated in LPS-treated cardiomyocytes, whereas SirT3-OE or AICAR decreased mitochondrial ROS accumulation (Figure 3D–3E). The levels of cellular anti-oxidants, including GSH and SOD, were decreased by LPS, which may prevent mitochondrial ROS clearance (Figure 3F–3G). However, SirT3-OE or AICAR increased the levels of GSH and SOD and thus reduced intracellular mitochondrial ROS (Figure 3D–3G).

Previous studies have proposed that damaged mitochondria may induce cardiomyocyte death by increasing the opening rate of mitochondrial permeability transition pore (mPTP) [37]. As shown in Figure 3H–3I, compared to the control group, LPS increased mPTP opening, which activated caspase-9. In contrast, SirT3-OE or AICAR treatment blocked mPTP opening (Figure 3H) and thus suppressed LPS-mediated caspase-9 activation (Figure 3I), suggesting that LPS may induce mitochondrial apoptosis in cardiomyocytes by inhibiting the SirT3-AMPK pathway.

### Mitochondrial biogenesis is increased by SirT3 via the AMPK pathway

**Figure 4:**
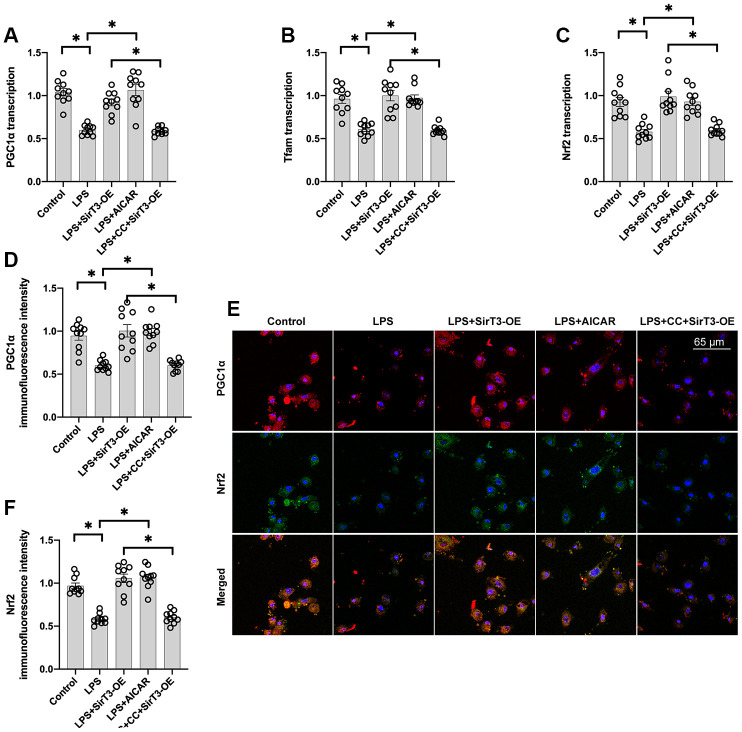
**Mitochondrial biogenesis is increased by SirT3 via the AMPK pathway.** (**A**–**C**) RNA was isolated from cardiomyocytes, and then transcription of mitochondrial biogenesis parameters was determined. Lentivirus-loaded SirT3 (SirT3-OE) and AMPK agonist (AICAR) were incubated with cardiomyocyte before LPS treatment. Compound C (CC), an antagonist of AMPK, was used to inhibit the activation of AMPK in SirT3-OE–transfected cardiomyocytes. (**D**–**F**) Immunofluorescence assay for Nrf2 and PGC1α in the presence of LPS. *P < 0.05.

Recent studies have found a relationship between SirT3 overexpression and mitochondrial biogenesis activation in kidney tumor cells [38] and osteoblasts [39]. Considering the beneficial effects exerted by mitochondrial biogenesis on mitochondrial turnover, we aimed to determine whether SirT3 induces mitochondrial biogenesis to preserve mitochondrial function under LPS stress. As shown in Figure 4A–4C, compared with the control group, genes associated with mitochondrial biogenesis, such as peroxisome proliferator-activated receptor-gamma coactivator 1 alpha (*PGC1α*), transcription factor A mitochondrial (*Tfam*), and nuclear factor erythroid 2-related factor 2 (*Nrf2*), were significantly downregulated at the transcriptional level in the LPS model. Interestingly, SirT3-OE or AICAR treatment increased mitochondrial biogenesis (Figure 4A–4C). To determine whether SirT3 modulates mitochondrial biogenesis via AMPK, compound C (CC), an inhibitor of the AMPK pathway, was added to SirT3-treated cardiomyocytes before LPS treatment. Compared to the control group, although SirT3 improved mitochondrial biogenesis (as demonstrated by increased *PGC1α*, *Tfam*, and *Nrf2*), this effect was negated with the addition of CC (Figure 4A–4C), confirming that AMPK is required for SirT3-activated mitochondrial biogenesis. Subsequently, immunofluorescence demonstrated that PGC1α and Nrf2 levels were significantly downregulated in LPS-treated cardiomyocytes (Figure 4D–4F). Although SirT3-OE upregulated PGC1α and Nrf2 expression, this action was nullified by CC (Figure 4D–4F). Our results demonstrate that SirT3 activates mitochondrial biogenesis through AMPK.

### Inhibition of mitochondrial biogenesis decreases SirT3-induced mitochondrial protection and cardiomyocyte survival

**Figure 5:**
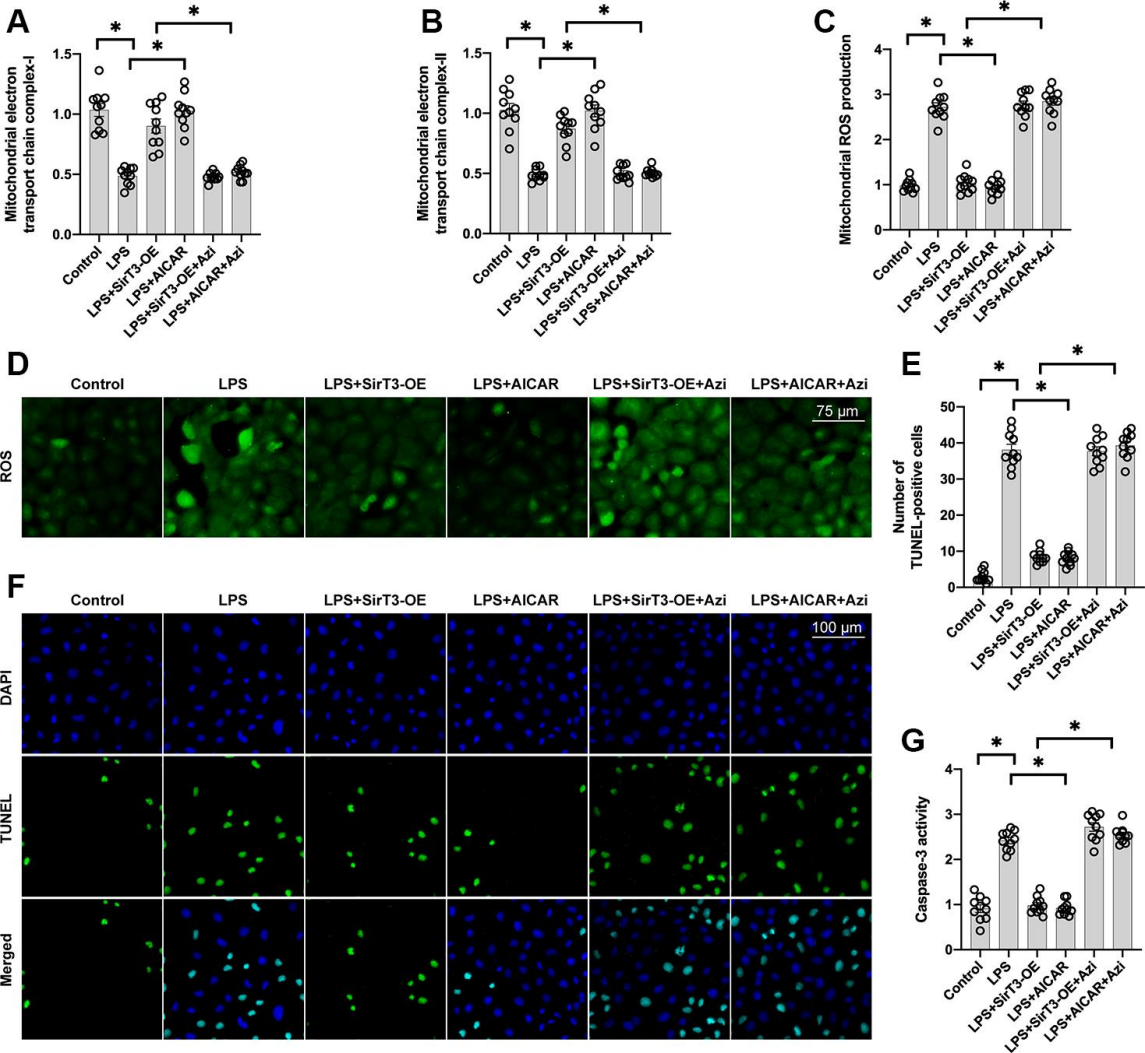
**Inhibition of mitochondrial biogenesis decreases SirT3-mediated mitochondrial protection and cardiomyocyte survival.** (**A**, **B**) ELISA assay was used to analyze mitochondrial electron transport chain complex activity in the presence of LPS. Lentivirus-loaded SirT3 (SirT3-OE) was incubated with cardiomyocytes in the presence of LPS. Compound C (CC), an antagonist of AMPK, was used to inhibit the activation of AMPK in SirT3-OE–treated cardiomyocytes. (**C**, **D**) Immunofluorescence of mitochondrial ROS production. (**E**, **F**) TUNEL staining of apoptotic cardiomyocytes. SirT3-OE was incubated with cardiomyocytes in the presence of LPS. CC was used to inhibit the activation of AMPK in SirT3-OE–treated cardiomyocytes. Then, the number of TUNEL-positive cardiomyocytes was determined. (**G**) ELISA was used to detect the activity of caspase-3. *P < 0.05.

To verify whether mitochondrial biogenesis is required for SirT3/AMPK-mediated mitochondrial protection, azithromycin, an antagonist of mitochondrial biogenesis, was used. Administration of azithromycin inhibited mitochondrial electron transport chain complex activity (Figure 5A, 5B) and induced mitochondrial ROS overload (Figure 5C, 5D) in cardiomyocytes treated with SirT3-OE or AICAR. Azithromycin was used to determine whether mitochondrial biogenesis activation is the underlying mechanism of SirT3/AMPK-induced cardioprotection, and TUNEL apoptosis staining and caspase-3 activity were measured. As shown in Figure 5E and 5F, compared to the control group, the number of TUNEL-positive cardiomyocytes was significantly increased after exposure to LPS. SirT3-OE reduced the ratio of apoptotic cardiomyocytes, but this effect was not seen in azithromycin-treated cardiomyocytes (Figure 5E, 5F). Similarly, caspase-3 activity was increased in cardiomyocytes when they were cultured with LPS (Figure 5G). Although SirT3-OE was shown to prevent caspase-3 activation, this effect was nullified by azithromycin (Figure 5G). Therefore, these results confirm that mitochondrial biogenesis, which is governed by the SirT3/AMPK pathway, protects against LPS-induced mitochondrial damage and cardiomyocyte death.

## DISCUSSION

Septic cardiomyopathy is a common feature of severe sepsis syndromes and is characterized by global ventricular dysfunction with decreased ejection fraction, diffuse cardiomyocyte death, and interstitial edema. Diagnostic approaches include echocardiography, detection of biomarkers such as troponin, and measurement of pro-inflammatory factors [40]. Several molecular mechanisms have been proposed to explain the pathogenesis underlying septic cardiomyopathy including cytokine-induced cardiomyocyte apoptosis, impaired microvascular reperfusion, histone-mediated angioedema, and mitochondrial dysfunction–related energy depletion [41]. In response to these possible pathological mechanisms, fluid resuscitation, vasoactive agents, negative chronotropic agents, and supportive care have been used to treat septic cardiomyopathy [42]. In the present study, we found that SirT3 downregulation and AMPK inactivation are the primary subcellular events in the progression of sepsis-induced myocardial damage. Decreased SirT3 was followed by AMPK inactivation, resulting in blunted mitochondrial biogenesis. Subsequently, defective biogenesis in mitochondria caused mitochondrial damage, as characterized by decreased mitochondrial metabolism, increased mitochondrial ROS production, and increased mitochondrial apoptosis. Mitochondrial dysfunction impaired cardiomyocyte contractility and even triggered cardiomyocyte death. Overexpression of SirT3 sustained mitochondrial function and cardiomyocyte viability through AMPK-controlled mitochondrial biogenesis. These findings provide new insight into the subcellular molecular mechanisms underlying septic cardiomyopathy. Based on our results, drugs targeting the SirT3-AMPK-mitochondrial biogenesis axis may benefit patients suffering from sepsis-related myocardial injury.

Recently, studies have examined the role of SirT3 in cardioprotection. For example, cardiomyocyte hypertrophy is improved by SirT3 activation via reduction of cytoskeletal protein expression [43]. Cardiomyocyte-specific overexpression of SirT3 reduces myocardial infarct size by inhibiting cardiomyocyte death and preventing infarct zone extension [44, 45]. With respect to arrhythmia, SirT3 upregulation stimulates Na^+^/K^+^-ATPase and thus moderately increases intracellular K^+^, augmenting the stability of cardiomyocyte membrane potential [46]. In addition, activation of SirT3 accelerates glucose metabolism via upregulation of PPARα [47], and SirT3-induced metabolic reprogramming has been shown to sustain cardiomyocyte function in a mouse model of heart failure [48]. Due to the critically important role played by SirT3 in cardiomyocyte contractility, metabolism, and survival, drugs targeting SirT3 have been developed and studied. For example, exendin-4, an anti-diabetes drug that has been used to control fasting blood glucose, upregulated SirT3 activity in a mouse model of myocardial ischemia-reperfusion injury [23]. Pyridostigmine, a vasoactive agent modulating blood pressure, has been reported to affect SirT3 expression and enhances glucose metabolism in diabetic mice [49]. Exogenous H_2_S, an anti-oxidative gas, interacts with SirT3 and promotes transcription of mitochondrial respiratory enzymes [50]. Unfortunately, no drugs targeting SirT3 have been investigated in the setting of septic cardiomyopathy. Thus, cellular and animal studies are needed to determine how to restore SirT3 in LPS-treated cardiomyocytes.

Mitochondrial biogenesis is significant in neonatal cardiomyocytes in order to meet the metabolic requirements of cardiomyocyte contractility [13, 51]. With increasing age, the extent and activity of mitochondrial biogenesis are slowly reduced [12, 52]. Therefore, mitochondrial biogenesis has been identified as a factor that may delay aging [53]. The goal of mitochondrial biogenesis is to produce new mitochondria through mitochondrial division, in order to sustain the mitochondrial cycle in cooperation with mitochondrial autophagy [54, 55]. Damaged or defective mitochondrial biogenesis is associated with an accumulation of old or dysfunctional mitochondria with reduced mitochondrial potential or increased ROS [56]. Subsequently, these damaged mitochondria disrupt cardiomyocyte metabolism and induce mitochondrial apoptosis or necrosis through mPTP opening [57, 58]. Therefore, activation of mitochondrial biogenesis is a protective tool to reduce cardiomyocyte vulnerability to stress. For example, increased mitochondrial biogenesis protects cardiomyocytes against ischemia-reperfusion injury by reducing mitochondrial oxidative stress and improving mitochondrial metabolism [59]. In addition, mitochondrial response sensitivity and mitochondrial senescence were shown to be normalized by mitochondrial biogenesis activation in a mouse model of myocardial hypertrophy [60, 61]. In this study, we observed the pathological alterations and protective mechanisms underlying mitochondrial biogenesis in septic cardiomyopathy. Our findings provide further evidence of the cardioprotective properties of mitochondrial biogenesis in cardiovascular disorders.

A limitation of our study is the lack of in vivo studies to support our findings. In addition, we did not evaluate the impact of the SirT3/AMPK axis on cardiomyocyte metabolism and the myocardial inflammatory response.

In conclusion, SirT3 downregulation, AMPK inactivation, and mitochondrial biogenesis inhibition are present in septic cardiomyocytes. Overexpression of SirT3 increases AMPK activity and improves mitochondrial biogenesis, which sustains mitochondrial function and reduces sepsis-related cardiomyocyte injury.

## MATERIALS AND METHODS

### Cell culture

Neonatal mice cardiomyocytes were isolated from 1- to 2-day-old mice. In brief, hearts were removed, ventricles were pooled, and cells were dispersed by successive enzymatic digestion with collagenase A (0.4 mg/mL, Roche) and pancreatin (0.5 mg/mL, Sigma-Aldrich) [62]. Cell suspension was thereafter purified by centrifugation through a discontinuous Percoll gradient to obtain myocardial cell cultures with 99% myocytes. After seeding on either plastic dishes coated with gelatin (0.2% in PBS, Sigma-Aldrich) or, for confocal microscopy, glass coverslips coated with poly-D-lysine (0.1 mg/mL, Sigma-Aldrich) in 30-mm plastic wells, cardiomyocytes were cultured in Dulbecco’s Modified Eagle Medium (DMEM)/medium 199 (4:1) supplemented with 10% horse serum, 5% calf serum, 1% glutamine, and antibiotics and placed in 37°C-5% CO_2_ atmosphere for 20 hours [63]. Approximately 95% of the cells displayed spontaneous contractile activity in culture. Then, cells, transiently transfected if necessary, were cultured in serum-free media for 24 hours before treatment. LPS was added into the medium to induce a septic cardiomyopathy model in vitro, as previously described [64].

### Lentiviral overexpression cell lines

SirT3 cDNA was synthesized as gene string from ThermoFisher Scientific and cloned in pLV lentiviral backbone. Lentiviral overexpression of SirT3 in cardiomyocytes was mediated by transduction of lentivirus from pLV plasmid carrying cDNA sequence of SirT3 and empty control [65]. For lentiviral production, HEK293T was transfected with pLV plasmid together with helper plasmids (vsvg, gagpol, rev, and NovB2) using CaCl_2_. Medium was changed after 8 hours of transfection. Viral supernatant was collected 48 to 72 hours later [66]. Cardiomyocytes were transduced with lentivirus, and after 3 days, puromycin selection was initiated to remove nontransduced cells. After puromycin selection, overexpression was confirmed by western blot [67].

### RNA isolation and PCR

RNA isolation from cell culture was done using Trifast (Peqlab) as per the manufacturer’s instructions. Isolated RNA (500-1000 ng) was reversed transcribed with random primer using iScript Select cDNA synthesis kit (Biorad). Real-time quantitative polymerase chain reaction (PCR) was done with iQ SYBR Green mix (Biorad) on C1000 Touch Thermocycler (Biorad) using specific primer pairs [68]. For amplification of circular RNAs, divergent primers were used, whereas normal linear transcripts were amplified by convergent primers as usual [69]. For RNase R resistant assay, RNA was incubated with RNase R (Biozym Scientific) at 37°C for 10 minutes and heat activated at 95°C for 3 minutes. RNA was then reverse transcribed and amplified by specific PCR primers as mentioned previously. Validation was performed as three individual experiments with three replicates each time [70].

### Mitochondrial function assay

Mitochondrial oxygen consumption and ATP synthesis rates were measured in saponin-permeabilized fibers using palmitoyl-carnitine/malate, pyruvate/malate, or glutamate/malate as substrate combinations, as described previously [71]. Mitochondria were isolated by differential centrifugation, and oxygen consumption and ATP synthesis were measured using palmitoyl-carnitine/malate, pyruvate/malate, or glutamate/malate as substrate combinations, as described previously [72]. Mitochondrial respiration complexes of isolated mitochondrial membranes were separated by blue native gel electrophoresis, and complex activities were determined by in-gel staining assays, as described previously [73]. Mitochondrial respiration rate in cardiomyocytes was assessed using a Seahorse XFp Extracellular Flux Analyzer with the XFp Cell Mito Stress Test Kit (Agilent, Santa Clara, CA) [74].

### MTT assay

MTT assay was performed using Cell Proliferation Kit I (Roche) as per the manufacturer’s instruction [75]. Briefly, cells were seeded in a 96-well plate and the next day treated with LPS in normal medium for 48 hours [36]. Next, 10 μL of MTT reagent were added, and cells were incubated for 4 hours followed by addition of dissolving reagent and incubation overnight. The next day, absorbance was measured at 580 and 690 nm by an HT Synergy (Biotek) plate reader.

### TUNEL staining

Cells were fixed with 4% paraformaldehyde for 20 minutes at room temperature and then permeabilized with ice-cold 0.1% Triton-X-100 in PBS for 2 minutes at room temperature. Next, cells were incubated with enzyme labeling solution provided with In Situ Cell Death Detection Kit (Roche) for 1 hour at 37°C. For negative staining, enzyme was not added to the labeling solution [76]. Cells were then washed and incubated with DAPI for 15 minutes. Images were taken with a Nikon Eclipse Ti microscope, and images were analyzed with Nikon NIS-Elements [33]. For analysis in each case, 10 different images were analyzed from different regions, and the average value was taken. Three data points representing three individual experiments are shown.

### Immunoblot analysis

Mitochondrial and cytosolic fractions were generated by homogenizing freshly excised hearts in homogenization buffer (20 mM HEPES, 140 mM KCl, 10 mM EDTA, 5 mM MgCl_2_, pH 7.4) with a Dounce tissue homogenizer, centrifuging the homogenate at 800 × *g* for 10 minutes and centrifuging the resulting supernatant at 8,000 × *g* for 10 minutes [77]. The supernatant is the cytosolic fraction. The pellet was washed by centrifugation at 10,000 × *g* and represents the mitochondrial fraction. Whole-cell extracts and mitochondrial membranes were prepared as described previously. Samples were loaded on SDS-PAGE, transferred to nitrocellulose or PVDF membranes, and incubated with specific antibodies [78]. Bands were visualized using horseradish peroxidase–conjugated secondary antibodies and the ECL detection system (GE Healthcare, Piscataway, NJ) or fluorophore-conjugated secondary antibodies and the Odyssey fluorescence detection system (Li-Cor Biosciences, Alpharetta, GA).

### Statistical analysis

Quantitative data are presented as mean ± standard error. Student’s t-test (two-tailed, unpaired) was used for comparisons between two groups; one-way and two-way ANOVA with Student-Newman-Keuls post hoc tests were used as appropriate to evaluate statistically significant differences in multiple group comparisons. *P* < 0.05 was considered statistically significant.
